# Production of mouse offspring from zygotes fertilized with freeze-dried spermatids

**DOI:** 10.1038/s41598-022-22850-5

**Published:** 2022-11-01

**Authors:** Sayaka Wakayama, Daiyu Ito, Masatoshi Ooga, Teruhiko Wakayama

**Affiliations:** 1 Faculty of Life and Environmental Science, University of Yamanashi, Kofu, 400-8510 Japan; 2 Advanced Biotechnology Center, University of Yamanashi, Kofu, 400-8510 Japan; 3 Laboratory of Animal Reproduction, Graduate School of Veterinary Science, Azabu University, Sagamihara, 252-5201 Japan

**Keywords:** Reprogramming, Animal biotechnology, Biotechnology, Developmental biology

## Abstract

Mouse cloning by nuclear transfer using freeze-drying (FD) somatic cells is now possible, but the success rate is significantly lower than that of FD spermatozoa. Because spermatozoa, unlike somatic cells, are haploid cells with hardened nuclei due to protamine, the factors responsible for their tolerance to FD treatment remain unclear. In this study, we attempt to produce offspring from FD spermatid, a haploid sperm progenitor cell whose nuclei, like somatic cells, have not yet been replaced by protamine. We developed a method for collecting FD spermatids from testicular suspension. Despite the significantly lower success rate than that of FD spermatozoa, healthy offspring were obtained when FD spermatids were injected into oocytes. Offspring were also obtained from FD spermatids derived from immature male mice that had not yet produced spermatozoa. These results suggest that nuclear protaminization, rather than haploid nuclei, is one of the key processes responsible for tolerance to FD treatment.

## Introduction

Preserving genetic resources is critical for preparing for the spread of unknown diseases or environmental changes such as global warming^1^. In mammals, spermatozoa or oocytes/embryos are cryopreserved in liquid nitrogen^2,3^. However, the use of liquid nitrogen has several drawbacks, such as high maintenance costs, and during a disaster, the liquid nitrogen supply may be interrupted, thereby destroying all genetic resources. To solve this problem, we developed a freeze-drying (FD) technique for mouse spermatozoa^4^. Although all spermatozoa died after FD treatment, their DNA remained intact, and those sperm acquired strong tolerance against temperature or radiation after FD treatment^5,6^. Healthy offspring can be obtained from FD sperm stored in a desk drawer^7^ or even on the International Space Station^6,8^ and from transported FD sperm on a postcard^9^. Notably, FD could be the best method for preserving genetic resources for a long time in a safe, low-cost, and location-independent manner^10–12^.

Because collecting spermatozoa from infertile animals, such as aged animals, animals with accidentally damaged reproductive organs, or immature males, is difficult, spermatids from testes containing haploid male germ cells may be collected before spermiogenesis is complete. Notably, although spermatids cannot fertilize oocytes by themselves, when spermatids are artificially injected into oocytes, healthy offspring can be obtained^13,14^. Despite the lower birth rates when this method is used than when mature spermatozoa are used^14,15^, spermatids are considered important components for preserving the genetic material of infertile or immature male animals^16^.

However, until recently, spermatozoa were the only type of FD cell from which offspring could be successfully produced. Owing to the large amount of cytoplasm that they contain, both oocytes and embryos are difficult to freeze-dry^17^. Although somatic cells or spermatids are the same size as spermatozoa, they have not been successfully used to produce offspring after being subjected to FD treatment for a long time^18–21^. Fortunately, we recently succeeded in generating cloned offspring from FD somatic cell nuclei, but the success rate was much lower than that of FD spermatozoa^22,23^. This could potentially be attributed to structural differences: The DNA of almost all somatic cell nuclei contains histones, whereas spermatozoa’s nuclei are haploid due to meiosis, and their histones are replaced by protamine during spermiogenesis, resulting in compact and hardened nuclei^24,25^. Therefore, haploid nuclei or nucleus hardening by protamine is required to overcome FD treatment-induced damage^26,27^.

This study’s aim is to clarify whether haploid nucleus or nuclear hardening by protamine is required to protect the nucleus from FD treatment effects. To this end, we attempt to generate offspring from FD spermatids. Although the success rate is extremely low, as with FD somatic cells, our findings provide a viable method of safely preserving the genetic material of infertile animals, even if power and liquid nitrogen supplies are interrupted during a disaster.

## Results

### Identification of freeze-dried round spermatid using a chromocenter

**Figure 1:**
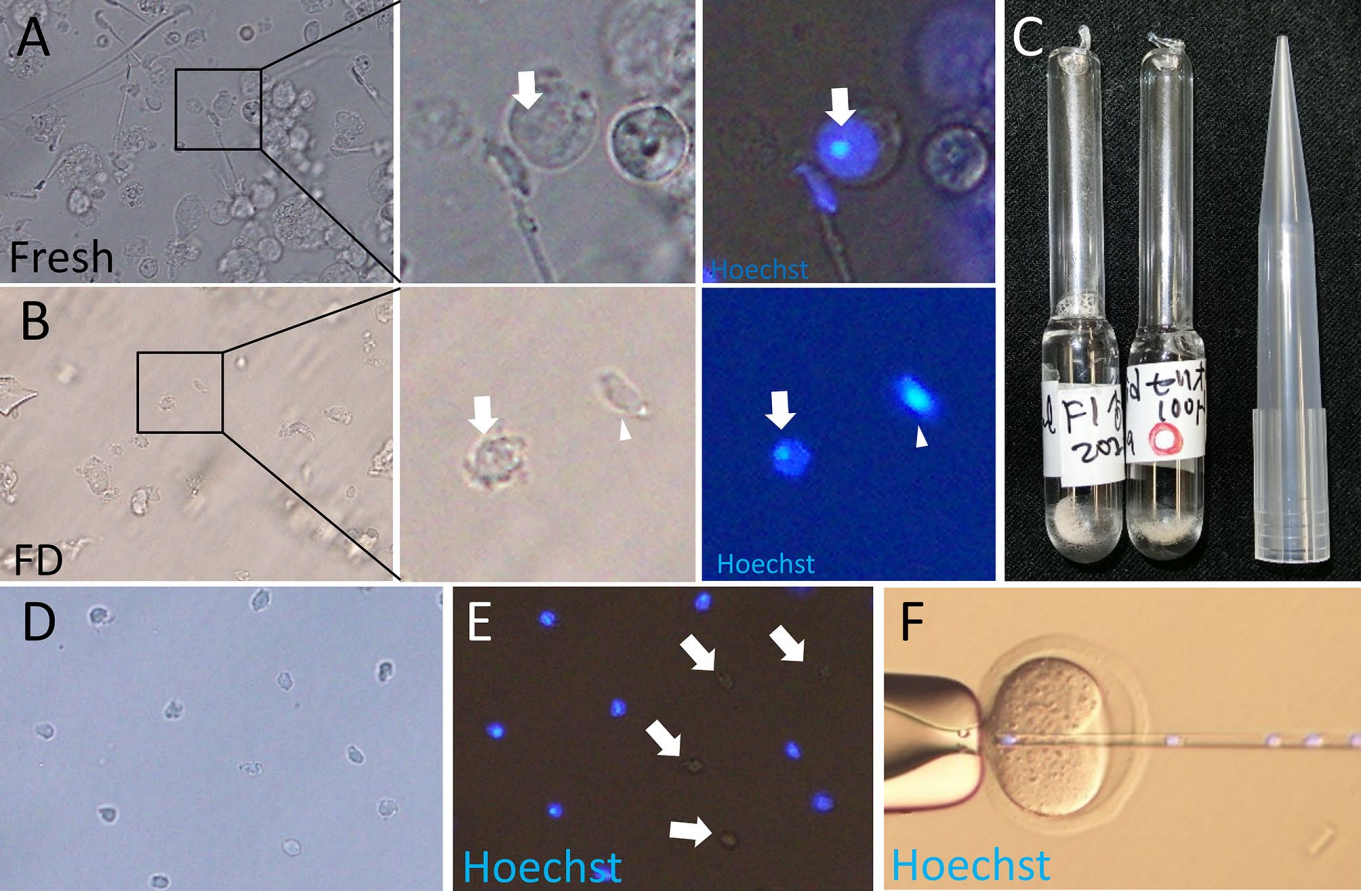
Detection of spermatids after FD treatment. (**A**) Fresh round spermatid. (**B**) Rehydrated FD spermatid. The middle section shows high magnification and the right section shows the fluorescent observation of the nucleus. The arrow shows the chromocenter. The arrow head shows an elongated spermatid. (**C**) Glass ampoules of mouse FD spermatids. (**D**, **E**) Collection of FD spermatids assessed as such morphologically (**D**), and fluorescent observation of FD spermatids (**E**). The arrow shows cells without nuclei. (**F**) FD spermatids were injected into fresh oocytes.

Round spermatids can be easily collected from fresh testicular suspensions using a glass pipette and micromanipulator, depending on the chromocenter in the nucleus (Fig. 1A), which is the unique and specific structure of round spermatids^28^. After FD treatment and rehydrating the testicular suspension (Fig. 1B, C), the membranes of many cells were destroyed, and many small debris remained. Although spermatozoa and elongated spermatids could be easily eliminated by their specific morphologies (Fig. 1B), the chromocenters of the round spermatids were not readily observable by light microscopy.

When these cell nuclei were stained with Hoechst 33342, the chromocenter of FD spermatids was readily observable, as was the case in fresh spermatids. Based on this observation, we noticed that the FD spermatid had a relatively small and clear cytoplasm and a relatively round morphology (Fig. 1B). Using such morphological markers, we tried to collect FD spermatids without fluorescent observation; as in offspring production, the toxicity/phototoxicity of the DNA-binding dye and the exposure of the cells to UV radiation must be completely avoided. However, in the first experiment, the selected cells, presumed to be round spermatids, were observed using Hoechst 33342 and UV light to check whether we could select perfectly round spermatids in this manner. Of the cells observed, 73% were classified as round spermatids owing to the chromocenter in the nucleus (Fig. 1D, E, Table 1). Some cells had no nuclei (18%). The remaining cells had flat and round nuclei (9%), suggesting that these cells were not single round spermatids but elongated spermatids.

**Table 1:** Detection of the chromocenter of cells collected by morphological markers using Hoechst staining.

Condition of spermatid	No. of examined cells	Nucleus was not existing in cytoplasm	Two nucleus in one cell	Chromocenter could not be observed	Detected the chromocenter
Fresh	105	2 (2)	2 (2)	0	101 (96)
FD	125	23 (18)	0	11 (9)	91 (73)

### Detection of the round spermatid-specific marker H3K9me3 in the zygote after ROSI

**Figure 2:**
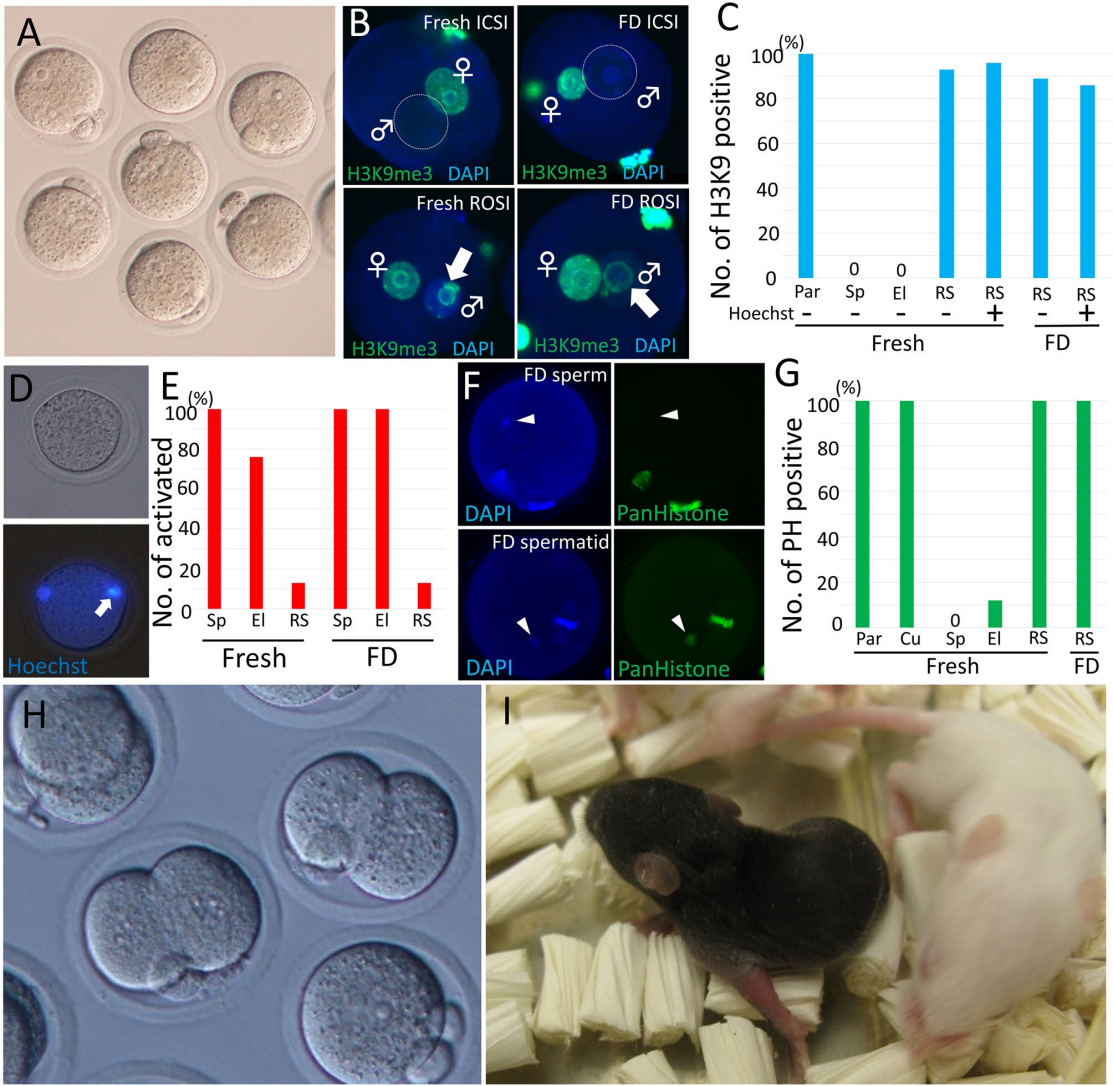
Confirmation of spermatid after injection into oocytes and production of offspring from FD spermatids. (**A**) Six hours after injection and activation, most oocytes formed male and female pronuclei. (**B**) Immunostaining of zygotes derived from fresh sperm (upper left), fresh spermatids (bottom left), FD sperm (upper right), and FD spermatids (bottom right) using the anti-H3K9me3 antibody. The arrow shows the male prenucleolar ring. (**C**) The ratio of H3K9me3 positive zygotes. Par: parthenogenetically activated zygote; Sp: spermatozoa; El: elongated spermatid; RS: round spermatid. The chromocenter of some round spermatids were observed before collection using Hoechst staining and UV light. (**D**) FD spermatids were injected into oocytes without artificial activation. Six hours after injection, these oocytes had pseudo-MII spindles derived from FD spermatid nuclei (upper, bright image; bottom, DNA under UV light). (**E**) The ratio of activated oocytes after germ cell injection. (**F**) Immunostaining of oocytes injected with FD sperm (upper) or FD spermatid (bottom). Left panel showed DNA staining by DAPI, and right panel showed histone staining using the anti-pan-histone antibody. The arrow shows the male pronucleus. (**G**) The ratio of pan-histone (PH) positive zygotes. (**H**) Two-cell stage embryos derived from FD spermatids. (**I**) Offspring were obtained from FD spermatids.

To confirm that the selected cells were round spermatids, they were injected into oocytes (Fig. 1F), and the male pronuclei (Fig. 2A) were examined using the round spermatid-specific epigenetic marker H3K9me3 (Fig. 2B)^29^. When fresh spermatozoa or elongated spermatids were injected into oocytes, and the male pronuclei of zygotes were immune-stained using an H3K9me3 antibody, none of them were stained. However, when fresh round spermatids were injected, most male prenucleolar rings (93–96%) were clearly stained with H3K9me3 (Fig. 2B, Table 2). Interestingly, 4–7% of the zygotes did not show a positive signal for H3K9me3, although we injected fresh round spermatids that were confirmed to have a chromocenter before injection. This suggests that some round spermatids were epigenetically not normal and could be one of the reasons for the lower success rate of offspring production from round spermatids than that from spermatozoa^14^. We then examined the male pronucleus of zygotes fertilized with presumed FD spermatids with or without confirmation of the chromocenter before injection into oocytes. The results showed that 86–89% of the zygotes exhibited positive signals for H3K9me3 in the male prenucleolar ring, irrespective of their chromocenter status before injection (Fig. 2B, C), suggesting that most of the selected FD cells were round spermatids and were even collected without Hoechst staining.

**Table 2:** Detection of H3K9me3 in the male pronucleus of zygote fertilized with different stages of germ cells with or without confirmation of chromocenter before ROSI.

Condition of cells	Cell type	Detection of chromocenter by Hoechst staining	No. of used oocytes	No. of activated oocyte	No. of two pronuclear formed zygote	H3K9me3 signal on the prenucleolar ring of male pronucleus	
						Positive	Negative
–	Oocyte*	−	20	20	20 (100)*	20 (100)*	0
Fresh	Spermatozoa	−	41	36	36 (100)	0	36 (100)
	Elongated spermatid	−	20	15	15 (100)	0	15 (100)
	Round spermatid	−	93	93	68 (73)	63 (93)	5 (7)
		+	70	70	52 (74)	50 (96)	2 (4)
FD	Round spermatid	−	100	93	57 (61)	51 (89)	6 (10)
		+	145	121	35 (29)	30 (86)	5 (14)

*Oocyte were parthenogenetically activated and female pronucleus were observed as control.

### Oocyte activation potential of injected cells

We also confirmed whether we might have accidentally injected FD elongated spermatids rather than round spermatids. Spermatozoa and elongated spermatids already contain sperm factors, such as PLC**ζ**, that activate the oocytes after fertilization/injection^30,31^. Because FD treatment does not cause loss of sperm factors^4^, the oocytes should be activated by sperm factors if FD elongated spermatids are injected instead of round spermatids. However, if round spermatids are injected, the oocytes should not be activated because sperm factors are absent in round spermatids. In the latter case, the nucleus of the injected spermatid will be condensed and form a spindle, which will show an MII-like structure inside the oocytes. As a result, when spermatozoa or elongated spermatids were injected, most oocytes were activated in either the fresh or FD treatments (Table 3). However, when the presumed FD spermatids were injected into oocytes, most oocytes failed to activate, and the nuclei of the injected round spermatids formed MII-like structures inside the oocytes (Fig. 2D), suggesting that these cells were actually spermatids. Although approximately 13% of oocytes were activated by the injection of presumed FD spermatids, a similar rate of oocytes was activated when fresh spermatids were injected (13%) (Fig. 2E, Table 3). This activation may cause the coinjection of sperm factors derived from lysed spermatozoa^32^.

**Table 3:** Oocyte activation potential of injected male germ cells of different stages with or without freeze-drying treatment.

Condition of cells	Cell type	No. of used oocytes	No. of survived oocytes after injection	No. of activated oocytes (%)	No. of pronucleus	
					1PN	2PN
Fresh	Spermatozoa	45	35	35 (100)	0	35 (100)
	Elongated spermatid	45	36	30 (83)	0	30 (100)
	Round spermatid	56	39	5 (13)	5 (13)	0
FD	Spermatozoa	42	32	32 (100)	1 (3)	31 (97)
	Elongated spermatid	62	57	57 (100)	1 (2)	56 (98)
	Round spermatid	150	135	17 (13)	17 (13)	0
Mock*	–	50	45	1 (2)	1 (2)	0

PN: pronucleus.

*For manipulation control, medium were injected into oocytes without nucleus.

### Detection of histones in FD spermatid-injected oocytes

Next, we examined whether the cell nucleus injected into oocytes had histones. If the cells we chose were spermatozoa or elongated spermatids in which histones were replaced with protamine, histones would be undetected in oocyte nuclei immediately after injection. However, if the cells we chose were spermatids, histones could be detected in the nuclei. When somatic cells (cumulus cells) as controls were injected into oocytes and immune-stained using pan-histone, all nuclei derived from cumulus cells and oocytes with MII spindles showed positive signals for pan-histones (Table 4). However, when fresh spermatozoa or elongated spermatids were injected into oocytes and immediately stained, although oocytes with MII spindles showed positive signals, 0% or 12%, respectively, of the injected nuclei showed positive signals (Fig. 2F, G). Then, fresh or FD spermatids were injected into the oocytes and immediately stained. In this experiment, all injected nuclei into oocytes showed positive signals, irrespective of fresh or FD conditions.

**Table 4:** Detection of pan-histone in the injected cell nucleus into oocytes.

Condition of cells	Cell type	No. of used oocytes	No. of oocyte without nucleus	No. of oocyte with pan-histone positive male nucleus
–	Oocytes*	20	0	20 (100)
Fresh	Cumulus	29	0	29 (100)
Fresh	Spermatozoa	46	0	0
	Elongated spermatid	24	0	3 (12)
	Round spermatid	63	0	63 (100)
FD	Round spermatid	89	19	70 (100)**

*MII spindle of intact oocyte were examined as positive control.

**This ratio was calculated against nuclear existed oocytes.

### Production of offspring from zygote fertilized with FD spermatids

Finally, we attempted to produce offspring from FD spermatids. When FD spermatids preserved at − 80 °C for 1–6 months were injected into oocytes, 76% of the zygotes developed into two-cell stage embryos the next day. The two-cell embryos showed a relatively normal morphology (Fig. 2H). After transferring the embryos into recipient females, ten offspring (3% from the transferred two-cell embryo) were successfully obtained (Fig. 2I, Table 5). Surprisingly, healthy offspring were obtained even from FD spermatids preserved for 1 year at − 80 °C (4%). The offspring success rate obtained from this method was significantly lower than that obtained from the use of fresh round spermatids (14%).

**Table 5:** Production of offspring from freeze-dried spermatids.

Age of male mice	Condition of spermatid	Storage duration	No. of oocytes that survived after injection	No. of activated oocytes	No. of embryos that reached the 2-cell stage*	No. of offspring (%)**	Average body weight (g)	Average placenta weight (g)
Adult	Fresh	–	152	108	106 (98)	15 (14)^a^	1.31 ± 0.16	0.18 ± 0.03
	FD	1–6 M	624	381	291 (76)	10 (3)^b^	1.74 ± 0.20	0.21 ± 0.05
	FD	1 Y	79	72	68 (94)	3 (4)^b^	nd	nd
3–4 W	Fresh	–	303	252	248 (98)	28 (10)^a^	1.34 ± 0.14	0.18 ± 0.06
	FD	1–6 M	538	514	409 (80)	3 (1)^b^	1.80 ± 0.06	0.18 ± 0.02

^a^ versus ^b^*P* < 0.05.

*All embryos were transferred into oviduct of recipient female.

**% from transferred 2-cell embryos.

We also tried to produce offspring from FD spermatids derived from 3- to 4-week-old immature male mice. These mice started spermiogenesis and only had round spermatids but did not produce spermatozoa in the testes. Therefore, if we could generate offspring from this experiment, there would be strong evidence that the offspring were actually produced from FD spermatids. When FD spermatids derived from immature males were injected into oocytes, three offspring (1%) were obtained. Although the success rate was lower than when fresh spermatids (10%) were used, these results clearly demonstrate that these offspring were generated from FD spermatids. The body and placenta weights of all offspring were within the normal range.

## Discussion

In the preliminary study, we attempted to select round spermatids by fluorescence-activated cell sorting before implementing FD treatment. However, we could not completely separate elongated and round spermatids, as suggested previously^24,33^. Then, we manually identified FD round spermatids using four different methods: morphology^14^, epigenetic markers^24,29^, oocyte activation potential^32,34^, and the existence of pan-histones^24^. In addition, we obtained offspring from zygotes fertilized with FD spermatids derived from immature males that are yet to produce spermatozoa in the testis^35,36^. Although these experiments offered indirect evidence, the results strongly demonstrated that we used FD round spermatids rather than elongated spermatids. In a few cases, we may have selected unknown cells instead of FD spermatids. However, based on their morphology, they were either somatic cells or secondary spermatocytes and never developed to the full term after being injected into oocytes. Unfortunately, the success rate of offspring production from embryos fertilized with FD spermatids was only 3–4%. Notably, the success rate of offspring production decreases significantly when FD spermatozoa are used compared with the use of intact spermatozoa (the average birth rate from fresh spermatozoa was around 60%, whereas from FD spermatozoa, it was around 20%)^7,37^. The success rate of offspring production also decreases significantly when fresh spermatids are used compared with when fresh spermatozoa are used (the average birth rate from fresh spermatozoa was around 60%, whereas, from fresh round spermatid, it was around 20%)^13,14,31^. Thus, the synergistic negative effect of both methods (FD treatment and the use of spermatids) would have greatly reduced the success rate of FD spermatids.

However, the production rate of offspring from the FD spermatid in this study was far too low. Because spermatids, like spermatozoa, are haploid cells, the lower success rate is most likely due to a lack of nucleus protaminization. Recently, we succeeded in producing cloned offspring from FD somatic cells, but the success rate was only 0.02%. These findings and those of the current study suggest that, although nucleus protaminization may not be essential since the offspring are born, nuclei protaminization is an important process in making the nucleus tolerant to FD treatment, either by nucleus hardening or by acting as a cryoprotectant.

Because it enables room temperature preservation, the use of FD sperm is a better solution than traditional cryopreservation for the long-term conservation of mammalian genetic resources. However, spermatozoa can only be collected from fertile males. This study revealed that genetic material from infertile or juvenile males can be preserved using FD spermatids. Although the offspring production rate was significantly lowered, as neither nuclear hardening nor protaminization is essential for FD treatments, preserving oocytes or embryos under FD conditions may be possible in the future. We have to develop a more stable and reliable method for FD spermatids, which may help develop a method for FD oocyte/embryos. The preservation of genetic resources at room temperature must commence before a serious disaster strikes Earth.

## Materials and methods

### Animals

BDF1 (C57BL/6 N × DBA/2; 4 weeks and 8–10 weeks of age) and ICR mice (8–10 weeks of age) were obtained from SLC Inc. (Hamamatsu, Japan). Surrogate pseudo-pregnant ICR females, which were used as embryo recipients, were mated with vasectomized ICR males, the sterility of which had been demonstrated previously. On the day of the experiment or after finishing all experiments, the mice were euthanized by CO_2_ inhalation or cervical dislocation and used for experiments. All animal experiments were conducted in accordance with the Guide for the Care and Use of Laboratory Animals and were approved by the Institutional Committee of Laboratory Animal Experimentation of the University of Yamanashi (reference number: A29-24), which followed the ARRIVE guidelines.

### Preparation of FD spermatids and FD spermatozoa

To collect spermatids, the seminiferous tubules of the testes were minced using sharp scissors, as described previously^14^; the only difference was that the cells were suspended in HEPES-buffered CZB medium^13^. In order to collect spermatozoa, we first collected epididymides and cut their ducts using sharp scissors, as described previously^31^. Aliquots (100 μL) of the testicular suspension or sperm suspension were dispensed into glass ampoules. The ampoules were kept at 4 °C for 3 h, − 30 °C for 3 h, and − 80 °C for several days until use. For the FD treatment, frozen ampoules were placed in liquid nitrogen for a few minutes and then freeze-dried using an FDU-2200 freeze dryer (EYELA, Tokyo, Japan). The cork of the freeze-dryer was opened for at least 3 h until all the samples were completely dry. After drying, the ampoules were sealed by melting the ampoule necks using a gas burner under vacuum, as described previously^5^.

### Oocyte preparation

Superovulation was induced in female mice via the injection of 5 IU of equine chorionic gonadotropin, followed by 5 IU of human chorionic gonadotropin (hCG) 48 h later. Cumulus–oocyte complexes (COCs) were collected from the oviducts of females 14–16 h later and moved to a Falcon dish containing an HEPES-CZB medium^38^. To disperse the cumulus cells, COCs were transferred to a 50 μL droplet of HEPES-CZB medium containing 0.1% bovine testicular hyaluronidase for 3 min. Cumulus-free oocytes were washed twice and moved to 20 μL droplets of a CZB medium^39^ for culture.

### Rehydration of FD spermatids or FD sperm and observation

Just before the round spermatid injection (ROSI) or intracytoplasmic spermatid injection (ICSI), the neck of the glass ampoule was broken, and 100 µL of sterile distilled water was immediately added and mixed using a pipette. For the observation of FD spermatids, 2 µL of suspension was mixed with Hoechst 33342 and placed in the manipulation chamber. Using microcapillaries, FD spermatids were collected and moved to different drops in the same chamber. Then, FD spermatids were observed using UV light to determine whether they contained a chromocenter.

### ROSI and ICSI

One to two µL of the rehydrated suspension was moved directly to the injection chamber. ROSI^40,41^ and ICSI were performed as previously described^31,38^. The oocytes that survived the ROSI/ICSI procedures were incubated in an CZB medium at 37 °C under 5% CO_2_ in humidified air. Pronuclear formation was checked 6 h after ROSI/ICSI.

### Oocyte activation and embryo transfer

For the ROSI experiments, oocytes were activated 15–20 min after the FD spermatid injection using 5 mM SrCl_2_ in Ca^2+^-free CZB medium for 1 h, followed by a culture in CZB medium until the embryo was used. The next day, fertilized embryos that had reached the 2-cell stage were transferred into the oviducts of pseudo-pregnant ICR female mice 0.5 days post coitum (dpc), which was when these mice had been mated with a vasectomized male the night before embryo transfer. On the day of embryo transfer, the recipients were anesthetized by an intraperitoneal injection (medetomidine, midazolam, and butorphanol). Between 5 and 8 embryos were transferred into each uterine horn, and an equal amount of atipamezole was injected. At 19.5 dpc, the offspring were delivered by caesarean section and randomly selected offspring were transferred to the cage of a foster mother who had delivered pups naturally. Three weeks later, the offspring were mated and their fertility was examined.

### Immunostaining of zygotes

Ten hours after the ICSI/ROSI, the zona pellucidae of surviving oocytes were removed using an acetic Tyrode solution, and the naked oocytes were fixed for 30 min at 25 °C in 4% (w/v) paraformaldehyde. The fixed oocytes were washed three times in PBS–polyvinyl alcohol (0.1 mg/mL PVA; Sigma-Aldrich, St. Louis, MO, USA) for 10 min and stored overnight at 4 °C in PBS supplemented with 1% (w/v) bovine serum albumin (BSA/PBS, Sigma-Aldrich) and 0.1% (v/v) Triton X-100 (Nacalai Tesque, Inc., Kyoto, Japan). The following procedure was previously described in^7^. The primary antibodies used were an anti-histone H3 (trimethyl K9) mouse monoclonal antibody (1:500; Abcam, Cambridge, UK) or an anti-pan-histone mouse monoclonal antibody (1:500: Merck, Darmstadt, Germany). The secondary antibodies used were Alexa Fluor 488-labeled goat anti-mouse IgG (1:500; Molecular Probes, Eugene, OR, USA) and Alexa Fluor 568-labeled goat anti-rabbit IgG (1:500 dilution; Molecular Probes). DNA was stained with 4′6-Diamidino-2-phenylindole (DAPI; 2 μg/mL; Molecular Probes). The brightness of the whole male pronucleus was measured using ImageJ and was then subtracted from the brightness of the zygote cytoplasm.

### Statistical analysis

Birth rates were evaluated using chi-squared tests. The statistical significance of any differences between the variables was set at *p* < 0.05.

## Data Availability

All data generated or analyzed during this study are included in this published article and are available from the corresponding author on reasonable request.
